# Comparative Evaluation of Physicochemical, Antioxidant, and Sensory Properties of Red Wine as Markers of Its Quality and Authenticity

**DOI:** 10.1155/2022/8368992

**Published:** 2022-10-17

**Authors:** Chigozie E. Ofoedu, Ebelechukwu O. Ofoedu, James S. Chacha, Clifford I. Owuamanam, Ifeyinwa S. Efekalam, Chinaza Godswill Awuchi

**Affiliations:** ^1^Department of Food Science and Technology, Federal University of Technology Owerri, PMB 1526, Owerri, Imo, Nigeria; ^2^Department of Food Science and Agroprocessing, Sokoine University of Agriculture, P.O. Box 3006, Chuo Kikuu, Morogoro, Tanzania; ^3^School of Natural and Applied Sciences, Kampala International University, Box 20000, Kampala, Uganda

## Abstract

The consumption of red wine by most wine drinkers has increased significantly because of the perceived health benefits which are linked to its inherent quality characteristics. In the quest to determine the conformity of Nigeria's domestic red wine quality with their international counterparts, the quality characteristics of domestic red wines produced in Nigeria were evaluated using foreign red wines as markers of wine quality and authenticity. Foreign and domestic red wines obtained in Nigeria were analyzed for physicochemical, antioxidant, and sensory properties using standard methods. Results showed that the domestic wines had significantly (*p* < 0.05) higher pH (4.03–4.16) and total sugar content (8.60–9.27%) while the foreign wines had significantly (*p* < 0.05) higher total titratable acidity (0.76–0.83%), Brix (6.98–8.04 g/100 g), alcohol (14.44–15.21% ABV), and polyphenol content (385.13–412.75 mg/L). Additionally, the domestic wines exhibited significantly (*p* < 0.05) lower antioxidant capacity compared to the foreign wines. Although the wines' hue angle (27.68°–41.46°) indicated a red colour spectrum in the visible region of the opponent colour chart, the total colour difference (Δ*E*) between foreign and domestic wines was significant. The sensory characteristics of the wines differed significantly as the panelist rating for overall acceptance ranged from 5.58 to 7.33. This research has provided valuable insight that the domestic wines studied showed a considerable level of authenticity and different levels of quality according to their varying concentration of organic acids, residual sugars, and phenolic compounds.

## 1. Introduction

Wines are originally alcoholic beverages obtained from total or partial fermentation of grape must or juice [1]. Wines are consumed for pleasure, and they are also popular for their ability to minimize the risk of heart disease due to the presence of antioxidants as well as extend life span (especially in red wines) when consumed moderately [2]. However, wines are not limited to grapes but can also be obtained from other sugar-containing plant fruits like mangoes, plums, pineapples, oranges, jackfruits, bananas, and elderberry and are generally referred to as fruit wines; fruits contain some of the phytochemicals and nutrients found in grape [3–5]. These fruit wines when produced are named after the parent fruit; for instance, wines obtained from pineapple fruits are known as pineapple wine [6] and failure to do so will be considered fraud.

Wines are of different grades and prices ranging from the very costly “Screaming Eagle Cabernet Sauvignon 1992” which was sold at $83,333USD (₦14,000,000) in the year 2000 to the cheap and not-so-great “Cocobon” red wine sold presently at $8USD (₦1,320) per bottle. The nature and quality of raw materials, terroir, processing technique, ageing etc. are some of the factors which influence the quality, value, and/or price of wines. According to England [7], wine has five basic characteristics which are major determinants of consumer acceptability/wine quality and a balance in these characteristics is what gives a good-quality wine. These are sweetness, acidity, tannin, alcohol, and body. Other factors that contribute to wine acceptability are colour and thickness (body/mouthfeel).

In the quest to make good affordable wines, winemakers are constantly developing means and ways of improving wine quality by using preferable good quality and expensive wines as markers or standards during production. This is done by varying the additives in different proportions to strike a balance among the basic characteristics to give a desired type of wine. This is not regarded as fraud since more than 60 additives can legally be added to the wine [8]. Winemakers, therefore, use these additives to tweak every wine characteristic be it colour, acidity, or thickness. Some of these modifications include reducing acidity with calcium carbonate, increasing acidity using tartaric acid, adding polysaccharides for a thicker mouthfeel, mass production of hundreds of gallons of wine in huge steel vats, infusing with oak chips to impart oaken barrel flavour, as well as using genetically engineered yeasts to produce certain flavours of wine [9]. Further, Bosker [8] revealed that major wineries all over the world employ this chemistry in their winemaking producing thousands of bottles that can even sell at the same price as bottled water.

In Nigeria, the perceived health benefits of red wines have increased red wine consumption and a slow decline in the consumption of other alcoholic beverages such as spirits and beer. [10, 11]. Nigeria was formerly listed among the top 30 countries in the world for wine consumption per capita, with roughly 10.57 liters drank per person [12–14]. Furthermore, European countries such as France, Spain, and Italy are the top importers of red wines to Nigeria, accounting for approximately 60% of total imports, while South Africa and the United States account for about 22% and 8% of the total imports, respectively [10, 15]. In tropical regions of the world for instance, in Nigeria, many wineries produce wines from locally available tropical fruits such as pineapple and mango, since grape is not grown in this part of the world.

The increased interest in the production of indigenous tropical red wine with the quest of finding an alternative to foreign red wines and saving the cost of foreign wine importation has provided insights into various practices in the utilization of locally obtainable materials for domestic red wine production. Further, while producing wine from tropical fruits, some manufacturers use artificial or natural colourants such as roselle calyces and flavourings, so that their product can compete favourably with foreign wines [16–20], while other manufacturers adulterate wines through the fraudulent act of blending different cheap ingredients (colourants, water, sugars, flavouring, and ethanol) to make wines. Given this, reputable wine industries, government agencies, and consumers are concerned about the quality and authenticity of wines in the markets. Specifically, wine quality is commonly evaluated based on sensory and chemical analyses [21]. Whilst colour, mouthfeel such as body and astringency, and taste (sweetness, sour, metallic, and bitterness) are used as wine quality indicators for sensory testing, the presence of phenolic compounds and other wine components are widely used for wine quality and authenticity assessment owed to fermentation effect [22–24]. This is partly because the phenolic compounds and other phytochemicals that are found in wine have been associated with many health benefits [25, 26]. There has not been a standard study or scholarly investigation of the wine quality (for instance, phenolic composition) sold in Nigeria, which will guard against fraud on the part of producers.

Additionally, the unanticipated economic contraction brought on by the COVID-19 pandemic resulted in economic instability, fast depreciation of the Nigerian currency, and high import costs for good-quality red wine [27]. This makes foreign red wines unaffordable to the average Nigerian. As a result, the consumers' attention has focused on affordable domestic red wines. On the other hand, due to the supply disruption of foreign red wines caused by the COVID-19 pandemic, lockdown, and related restriction measures [28], local manufacturers capitalized on this opportunity and established ways to increase and sustain the production of domestic red wines. As consumers' attention was diverted to the consumption of domestic red wines, the answer that they thought they had found in these affordable domestic red wines proved to be fleeting, as it was discovered that many of these wines tasted nothing like the good foreign red wines. This makes one wonder if the so-called “domestic red wines” found on the shelves in wine shops are wine, hence the need to analyze these wines using their foreign counterparts as markers of quality.

It is therefore important to carry out a quality evaluation of red wines produced and sold within Nigeria to ascertain their level of authenticity and whether they meet the quality characteristics of their international counterparts in the market. Understanding the quality characteristics of domestic wines in relation to foreign wines will guide producers on raw material selection for optimized output while putting into consideration the preference and health status of the consumer. The information obtained from this research work will identify lapses in the quality characteristics of domestic wines, especially from wine producers, as well as highlight how the quality of most domestic wines can be improved upon. This research work will create awareness of the quality attributes of different Nigerian wines in the market and thus guide regulatory agencies in setting standards for Nigerian-made wines. In this context, therefore, the objective of this research was to evaluate the physicochemical, antioxidant, and sensory properties of red wines sold in Nigeria to determine their quality and authenticity.

## 2. Materials and Methods

### 2.1. Schematic Overview of the Experimental Study

**Figure 1:**
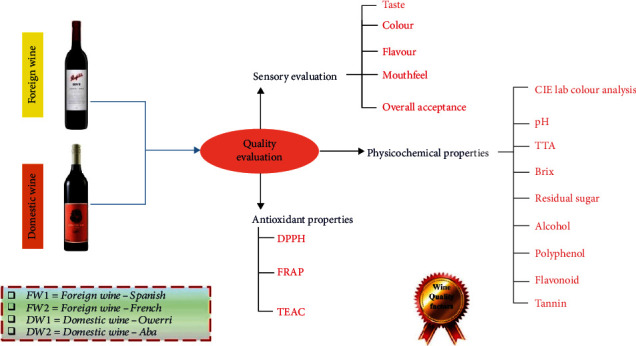
Schematic overview of the experimental study.

The experimental program depicts the diagrammatic synopsis of crucial stages from procurement of red wine samples to laboratory analyses of wines as shown in Figure 1. Specifically, this study was designed to determine some quality characteristics of different (foreign and domestic) wines sold in southeast Nigeria by evaluating their physicochemical, antioxidant, and sensory properties.

### 2.2. Sample Procurement

A total of 14 red wine brands used in this current study were obtained from wine shops in the cities of Aba and Owerri, both in southeast Nigeria. Specifically, ten wine samples were foreign red wines (five each from Spain and France) while the other four wine samples were domestic red wines (two each from Aba and Owerri). Importantly, wine selection was done based on popularity and use. Prior to wine quality evaluation, wine samples were prepared and grouped into four categories, namely, foreign wine—Spanish (FW1), foreign wine—French (FW2), domestic wine—Owerri (DW1), and domestic wine—Aba (DW2). The results of any categorized group (i.e., FW1, FW2, DW1, or DW2) for an evaluated parameter were determined by averaging the data obtained from each subset in that group to give its mean value.

### 2.3. Evaluation of Physicochemical Properties of Wine Samples

#### 2.3.1. Determination of Wine pH and Apparent Brix

The pH of the wine was determined using a digital pH meter according to the method described by AOAC [29]. The digital pH meter was calibrated using different buffer solutions (4, 7, and 9) at 25°C. Measurement was done by immersing the pH electrode (probe) into a 25 mL pipetted wine sample and was allowed to stabilize before reading off.

On the other hand, the wine Brix was determined using a Digital Brix Refractometer Model MA871 (Milwaukee Instruments, NC, USA) as described by Montañez-Soto et al. [30]. The refractometer was standardized with distilled water at 20°C until the Brix value reads zero. Subsequently, two drops of wine sample were placed on the lens (sensitive surface) and measurement was taken afterwards.

#### 2.3.2. Determination of Wine Total Titratable Acidity (TTA)

The TTA was determined according to the method described by AOAC [29]. A known volume of the wine sample (15 mL) was diluted with distilled water (10 mL) and then added to a conical flask, followed by additions of 3 drops of 5% phenolphthalein as an indicator. The mixture was homogenized to ensure proper mixing and titrated with 0.1 N NaOH against a white background until the solution turns pink and retained its colour. The TTA was expressed as % tartaric acid and calculated using the equation as follows:

(1) $$\begin{array}{c} \text{Total titratable acidity}=\frac{V\times N\times 75}{v\times 1000}\times 100, \end{array}$$

where *V* = milliliters of NaOH solution used for titration, *N* = normality of NaOH solution, and *v* = wine sample volume.

#### 2.3.3. Alcohol by Volume

The method for determination of alcohol by volume (%) was done according to the procedure described by Onwuka [31] through the distillation technique. The wine sample (100 mL) was measured out into a volumetric flask at 20°C. The sample was washed into a distillation flask using 50 mL of distilled water. The mixture in the distillation flask was placed over a heating mantle and distilled, followed by cooling at 20°C to collect the alcohol as distillate. The volume of the distillate recovered was measured and the alcohol yield was determined as % ABV which is calculated as follows:

(2) $$\begin{array}{c} \%\text{alcohol by volume}=\frac{\text{volume of distillate}}{\text{volume of sample used }}\times 100. \end{array}$$

#### 2.3.4. Determination of the Total Sugar Content

The sugar content of wine samples was measured using the phenol-H_2_SO_4_ method as described by Nielsen [32] which involves calculating the sugar content of wine using the calibration equation *y* = 0.0127*x* + 0.0131 at *R*^2^ = 0.996, where *y* and *x* stand for absorbance reading and sugar concentration expressed in g/L equivalent to dextrose glucose, respectively.

#### 2.3.5. CIE Colour (*L*^∗^, *a*^∗^, and *b*^∗^) Analysis of Wine

The CIE colour (*L*^∗^, *a*^∗^, and *b*^∗^) values were measured using a Hunter LabScan Spectrophotometric colorimeter controlled by a computer that calculates colour coordinates from the reflectance spectrum as described by Osuji et al. [33]. Reflection of samples in wavelength domains of 400 to 700 nanometers was measured. The colour factors were calculated under a lightness of D65 and visual angle of 10^o^. To calculate the colour difference and for a more precise evaluation of wine colour changes in different samples, the amount of colour difference between the wine samples (Δ*E*^∗^), angle of hue (*h*°), and colour purity (Chroma) (*c*) was calculated.

#### 2.3.6. Determination of Flavonoids

The total flavonoid content of wine samples was determined using the UV-VIS spectrophotometric method at a wavelength of 510 nm as described by Ahmed et al. [34]. Subsequently, the flavonoid content was calculated using the calibration curve/formula, *y* = 0.0236*x* + 0.0348, where *y* is the absorbance at 510 nm and *x* is the amount of concentration in *μ*g/mL. The flavonoid content was calculated as catechin equivalent (CE) in milligrams per liter of wine.

#### 2.3.7. Determination of the Phenolic Content

The phenol content of wine samples was determined using the Folin-Ciocalteu colorimetric method with the aid of a UV-VIS Spectrophotometer Model 6305 (Bibby Scientific Ltd., UK) at a wavelength of 760 nm as described by Singleton et al. [35]. The total phenolic content (TPC) was expressed as milligrams of gallic acid equivalents (GAE) per liter of wine.

(3) $$\begin{array}{c} \text{Total phenolic content}=\frac{C\times V}{W}, \end{array}$$

where *C* = concentration of gallic acid calculated from the calibration curve (mg/mL), *V* = volume of the sample (mL), and *W* = weight of the sample (g).

#### 2.3.8. Determination of Tannin

The tannin content of wine samples using the methyl cellulose precipitation method was described by Sarneckis et al. [36] with the aid of a UV-VIS Spectrophotometer Model 6305 (Bibby Scientific Ltd., UK) read at an absorbance of 280 nm. Further, the amount of measured tannin expressed in % was calculated using the equation as follows:

(4) $$\begin{array}{c} \text{Tannin }\left(\%\right)=\frac{A_{u}\times C\times V_{f}Vf\times 100}{W\times A_{s}\times V_{a}}, \end{array}$$

where *V*_*f*_ = total volume of sample, *V*_*a*_ = volume of sample, *A*_*u*_ = absorbance of the test sample, *A*_*s*_ = absorbance of standard solution, *W* = weight of sample used, and *C* = concentration of standard solution.

### 2.4. Antioxidant Properties of Wine

#### 2.4.1. DPPH Radical Scavenging Activity

The DPPH radical scavenging activity of wine samples was determined according to the method reported by Yang et al. [37] with slight modifications. Briefly, 0.1 mL of 0.2 mmol/L freshly prepared DPPH solution was added to 0.1 mL of the sample and homogenized; then, the mixture was incubated at room temperature for 30 minutes in the dark. The absorbance of the mixture was measured at 517 nm. DPPH radical-scavenging activity is expressed as % and determined as the following formula:

(5) $$\begin{array}{c} \text{DPPH radical scavenging activity}\left(\%\right)=1–\frac{A1-A2}{Ao}\times 100, \end{array}$$

where *A*1 = absorbance of the wine sample, *A*2 = absorbance of absolute ethanol instead of DPPH solution, and *A*0 = absorbance of solvent instead of the sample solution.

#### 2.4.2. Ferric Reducing Antioxidant Power (FRAP) and Trolox Equivalent Antioxidant Capacity (TEAC)

The ferric reducing antioxidant potential (FRAP) of the wine sample was measured according to the method proposed by Benzie & Strain [38]. The absorbance of the mixture (FRAP reagent and wine sample) was measured at 593 nm. The results were reported as *μ*g of ascorbic acid equivalents (AAE) per mL.

However, the Trolox equivalent antioxidant capacity (TEAC) assay was determined according to Ozgen et al. [39]. The absorbance of the mixture was measured at 734 nm. The Trolox calibration curve was plotted as a function of the percentage of ABTS radical cation scavenging activity. The final results were expressed as mmol of Trolox equivalents per liter of the sample (mmol/L).

### 2.5. Sensory Evaluation of Wine Samples

The hedonic scale method was employed to evaluate the sensory qualities of wine samples as described by Iwe [40]. This entailed comparing domestic wines to foreign wine samples. Twenty regular wine drinkers (semitrained panelists) of various ages (20–36 years) with postsecondary education participated as tasters in the sensory evaluation. The necessary criteria for taking part in the study were that the individual consumed wine and expressed interest in participating in all test sessions. Importantly, participation in this sensory evaluation was entirely voluntary, with prior oral agreement obtained. At temperatures of around 10°C, the coded wine samples were served at random. The tasters were given twenty series of wine samples in transparent wine glasses, and their degree of preference was assessed using a nine-point hedonic scale, with 9 indicating extremely liked and 1 indicating extremely disliked, for five main attributes: aroma, mouthfeel, taste, and appearance. However, the overall acceptance of the samples was evaluated by averaging the other attributes. To eliminate crosscontamination between samples, the panelists drank potable water to rinse/clean their mouths between tastings. The tasters completed score sheets after each tasting.

### 2.6. Statistical Analysis

Data obtained from duplicate measurements were subjected to one-way analysis of variance (ANOVA) using IBM SPSS version 24 Software (IBM, New York, USA). Fisher's least significant difference (LSD) was used to statistically resolve the significant (*p* < 0.05) mean difference amongst the measured parameters which were expressed as the mean ± standard deviation (SD).

## 3. Results and Discussion

### 3.1. Variations in Physicochemical Properties of Wine Samples

#### 3.1.1. pH

The pH of wine samples ranged from 3.51 to 4.16 (refer to Table 1). The foreign wines had pH levels of 3.51 and 3.87 while the domestic wines had a pH of 4.03 and 4.16. There were significant differences (*p* < 0.05) between the wine samples. All wines appear to be in the acidic region. pH is well known as the strength or measure of the degree of relative acidity against the relative alkalinity of any liquid [41]. The pH of foreign wines was significantly (*p* < 0.05) lower than the pH of domestic wines. The variations in pH levels could be a result of the type and variety of fruits used, their degree of ripeness, type of soil, and processing conditions (fermentation and ageing) as well as methods [42]. According to Schulfer [43], the average pH levels for red wines are between 3.50 and 3.80. In the same vein, the pH levels of blends of pineapple-carrot wine produced by Balogun et al. [44] were also in the range of 3.20 to 3.50.

Further, the result of the current study shows that the pH levels of foreign wines fell within this region while that of domestic wine was slightly above the 3.20–3.80 region of red wines. The higher pH levels in the domestic wines (4.03–4.16) could be attributed to differences in the fermentation substrate (must) and incomplete fermentation process [45] such as stuck fermentation. Additionally, the lower pH level in foreign wines might be due to higher concentrations of acids (tartaric and succinic acid) in the wine compared to domestic wines. Besides low wine pH depicting sharpness (tart and crisp) in taste [41, 46], it also plays a critical role in wine quality and stability (shelf life) [47]. Whilst higher pH wines are more susceptible to bacterial growth, low pH wines between 3.20 and 3.80 are desirable because they discourage bacterial growth and spoilage [48].

#### 3.1.2. Total Titratable Acidity (TTA)

The TTA of wine samples ranged from 0.56 to 0.83% (refer to Table 1) with the foreign wines having a TTA of 0.76% and 0.83% while those of domestic wines were 0.56% and 0.68%. There were significant differences (*p* < 0.05) between the TTA of the red wine samples. TTA is among the commonly determined wine quality parameters because challenges of acidity are among the key hurdle food processors which are confronted with [49, 50]. Results show that foreign red wines had a higher TTA (0.76–0.83%) compared to domestic red wines with a TTA of 0.56–0.68%. The TTA of wines are within the range of 0.65% for date palm fruit wine [51], higher than the value of 0.47% for pineapple-banana wine [52] and lower than the value of 2.30% for mixed fruit wine [53]. According to Chilaka et al. [54], the total acidity of a wine is expected to range from 0.5 to 1.0%. The variations in wine TTA could be a result of differences in the type and variety of fruits used as fermentation must and the nature of alcoholic fermentation. Conde et al. [55] reported that organic acid concentrations in grape musts are predominantly a function of grape maturity and variety, and the same phenomena may apply to other fruits. On the other hand, alcoholic fermentation influences the concentration and content of wine acidity and may result in different wine acidity levels [56–58]. The acid content in red wine is of great importance to the preservation (wine stability) in terms of shelf life extension and sensory characteristics of wine. Besides acidity affecting the wine taste, the colour quality of red wine is also influenced by its TTA [42].

TTA is influenced by the presence of more hydrogen ions either attached to organic acids or in form of free ions, and this may give these wines their distinctive taste and flavour [59, 60]. Furthermore, the lower TTA in domestic red wines could be due to the lower concentrations of organic (citric, malic, succinic, propionic, butyric, tartaric, etc.) acids influenced by yeast strain and fermentation conditions. Furthermore, wine contains numerous organic acids that are volatile and contribute to the acidity of the entire wine. Acetic acid, for example, is the predominant volatile acid found in wine as a result of fermentation despite its small concentration [61]. In addition to the acids, other acids found during wine fermentation include but are not limited to acetic, lactic, glucuronic, galacturonic, and phenol-carbonic acids (which affect wine taste and colour) [42]. Notably, there is an inverse relationship or correlation between TTA and wine pH as foreign red wines with higher TTA had lower pH while the domestic red wines with lower TTA had higher wine pH.

#### 3.1.3. Total Sugar

The total sugar content of wine samples is presented in Table 1. The total sugar content ranged from 6.13 to 9.27% and showed significant differences (*p* < 0.05) between the wine samples, amongst the total sugar content and reducing sugar content. Overall, results show that foreign wines which recorded a lower total sugar content had higher TTA (refer to Table 1) compared to the domestic wines. It is generally known that the sugar content and TTA correlate inversely because during ripening, as sugar levels tend to increase, their acidity levels decrease. However, about the type of wine, the higher sugar content in domestic wines when compared to the foreign wines in this study could be due to the differences in agronomic practices and climate conditions. Fruits for instance grapes, from cooler (temperate) regions, generally have higher levels of acidity due to the slower ripening process, compared to fruits from warmth (tropical) regions with more available sunshine which increases fruits' sugar content and pH (lower acidity level) [62].

Sugars are the main source of perceived sweetness in the wine, and they come in several forms. Total sugars, a measure of the residual sugars (sugars remaining in the wine after the completion of fermentation) in this context, are a combination of fermentable and nonfermentable sugars. The foreign wine samples had a relatively lower amount of total sugars compared to that of domestic wines. These variations may be attributed to the type and/or variety of fruit used and yeast strain. Nonfermentable sugars such as cellobiose, galactose, pentoses, and arabinose contribute to the total amount of sugars found in wine but may offer little or no sweetness to wines due to their negligible amount and also possess no sweet taste to humans [63]. On the other hand, glucose, fructose, and sucrose are the predominant fermentable sugars found in red wines which also contribute significantly to the sweetness of the wine. Fermentation generally reduces the sugar content of wines to between 0.5% (dry wine) to 3.0% (sweet wine) [61]. The total sugar content of red wine in this study is above the sugar content of less than 0.4% for dry table wine but within the range of 6–16% for liqueur wine reported by Gnilomedova et al. [64].

However, the foreign wine samples had a relatively lower amount of reducing sugars compared to the domestic wines. These variations may be attributed to the type and/or variety of fruit used and yeast strain. Amongst the reducing sugars (glucose, fructose, and pentose) found in red wine, glucose is preferentially utilized by yeast because of its molecular weight, and as a result, the residual sugar which is an indication of the total sugars remaining after fermentation is typically composed of about 60–70% fructose [63]. As a result of the selective transport of sugars by yeast strain during fermentation, most unutilized sucrose in the wine eventually undergoes inversion to yield an equal amount of glucose and fructose [63], thus contributing to the overall fructose content in wines. This was evident in the work of Gnilomedova et al. [64] who reported higher fructose content in semisweet table wine and liqueur wine. Notably, fructose is known to taste twice as sweet as glucose because of its unique interaction with the taste bud receptors.

#### 3.1.4. Brix

The Brix value of wines changed significantly (*p* < 0.05) and varied between 4.22 and 8.04 g/100 g (refer to Table 1). Specifically, foreign wines had a higher Brix value (6.98–8.04 g/100 g) after fermentation compared to domestic wines with a Brix value of 4.22–5.08 g/100 g. The lower Brix values of domestic wines when compared to foreign wines may not principally be an indication of the efficiency of the fermentation process in terms of yeast utilization of soluble extracts to produce ethanol and other compounds. However, the variations and significant differences (*p* < 0.05) amongst the apparent Brix of wine samples could be attributed to the inherent differences among the fruits used and the nature of fermentation. The type and amount of sugars in the wine after fermentation can affect the Brix measurement as a result of changes in the refractive index of the solution due to the different soluble substances in the wine samples [59, 65, 66]. A Brix refractometer measures the degree to which a solution refracts or bends light, as it is normally used to measure the amount of sucrose in a solution [67, 68].

Interestingly, for the wine samples to have Brix values after fermentation, is an indication of either a fermentation problem such as stuck fermentation or that the wine samples did not ferment to dryness [69], probably due to the type/style of wine that the winemaker intends to make. In addition to Brix being a relatively good measure of the total sugar content, a positive correlation and a satisfactory quality of fit (*R*^2^ adjusted) between Brix value and total sugar have been reported in several studies [70]. On the contrary, the relationship between Brix and total sugar content in this study appears to be inversely related. This could imply that other compounds might have contributed to the wines' Brix values besides sugar alone. It is well known that Brix is a measure of density. Given this, all kinds of soluble substances can affect wine Brix values [63]. Additionally, besides sugars, the number of dissolved solids such as vitamins, minerals, amino acids, organic acids, polyphenols, tannins, and colour substances might also contribute to influencing the Brix values of the wines. This corroborates the findings of Chiralt and Martinez-Navarrete [71] that Brix helps to validate the presence of soluble solids, especially for non-sucrose-based products.

#### 3.1.5. Alcohol Content

The alcohol content of wine samples varied significantly (*p* < 0.05) ranging from 8.52 to 15.21% ABV (refer to Table 2) with the foreign wines having an alcohol content of 14.44% ABV and 15.21% ABV while those of domestic wines were 8.52% ABV and 10.68% ABV. Alcohol is present in wines because of the fermentation of carbohydrates (mainly sugars) by yeast. In this current study, the foreign wines recorded higher alcohol content when compared to the domestic wines. The variations in alcohol content could be a result of differences in sugar concentration, type of sugars, the strain of yeast, and fermentation conditions. It is well-known that among other factors, the sugar quality and concentration influence the mode of sugar utilization by yeasts during fermentation. Besides shaping the product quality, the management of alcoholic fermentation is crucial to wine stability over time [72]. Further, ethanol is the most predominant alcohol found in fermented beverages, especially wines. It is a product of the glycolytic pathway, having pyruvate as the intermediate compound, which is then decarboxylated into acetaldehyde, followed by reduction to ethanol [73]. However, ethanol does not only give off an alcoholic aroma and body to wines but it also acts as a vehicle for other aroma-active volatile compounds [74]. As the only alcohol generally present in sufficient amounts, ethanol presents an important sensory attribute in wines as it produces complex sensory perceptions that possess the distinctive aroma, activates the perception of sweetness, and stimulates the sensations of heat and weight in the mouth [73].

The alcohol content of domestic wines (8.52–10.68% ABV) compares favourably with 5.5–9.0% ABV reported by Awe and Nnadozie [51] and 8.1% ABV reported by Ajit et al. [52]. In this study, the foreign wines and domestic wines can be categorized as medium-bodied wine and light-bodied wine, respectively, according to their alcohol level and the nature of the impact on the palate. According to Richards [75], wines are categorized as low-alcohol or low-bodied (6–11% ABV), medium-bodied (12–16% ABV), and full-bodied (17–23% ABV) wines. Interestingly, results show that the sugar profile and its concentration significantly influenced the alcohol content of wines. Domestic wines with higher residual sugars had lower alcohol content while foreign wines with lower residual sugars had higher alcohol content. Besides the sugar profile of must, the differences in their alcoholic content could also be attributed to incomplete or stuck fermentation that prevented optimal utilization of sugars in the must [76]. Also, concerning foreign wines having higher alcohol content with higher Brix value and vice versa, this suggests that alcohol concentration in wine does not solely depend on the sugar profile of must, but more likely on the available fermentable extracts (nitrogenous compounds, mineral, etc.) readily utilized by yeasts [46, 77].

In addition to ethanol, fusel alcohol (also known as fusel oil or higher alcohol) are important alcohols that impart sensory attributes in wines. They are the major volatile by-products of fermentation biosynthesized by yeasts. Fusel alcohols include but are not limited to propanol, isobutyl alcohol, isoamyl alcohol, hexanol, phenethyl alcohol, and pentyl decanol that possess flavourful organoleptic attributes such as being fruity, alcoholic, pungent, solvent-like, rose-like, or floral which contributes to the overall sensory quality of wine from the total alcohol content [73].

#### 3.1.6. Polyphenol Content

The polyphenol contents of wines ranged from 216.73 to 412.75 mg/L (refer to Table 2) with the foreign wines having a polyphenol content of 385.13 mg/L and 412.75 mg/L while those of domestic wines were 216.73 mg/L and 306.42 mg/L. There were significant differences (*p* < 0.05) between the polyphenol content of the red wine samples. Polyphenols are the most abundant and important phytochemical in red wine [78, 79]. It is the main bioactive compound found in wines [80]. Specifically, foreign wines had higher polyphenols (385–412.75 mg/L) compared to domestic wines (216.73–306.42 mg/L). The variation in the polyphenolic composition of the wine could be due to differences in the type of fruit (for instance, grape) used, its varietal difference, the agronomic (vinification) practice, environmental (soil, climate, amount of sunlight, etc.) conditions, fruit maturity stage, vinification process, storage duration of wine, type of yeast used in the fermentation, and whether fruit solids are present in the maceration process [81–83]. Also, fruit location and production year could cause a significant difference in the amount of polyphenolic content in wine.

Red wine contains different types of polyphenolic compounds in various concentrations, most of which come from the skin and seeds of fruit during the fermentation process [81]. The presence of ethanol and its solvent properties as a hydrophilic compound facilitates the polyphenol extraction process into wines [84, 85]. However, polyphenols are categorized as flavonoids (anthocyanins, flavan-3-ols, proanthocyanidins, and flavanols) and nonflavonoids (phenolic acids and stilbenes) [82, 86]. Interestingly, polyphenols are responsible for some sensory characteristics in red wine such as the wine's colour, bitterness, and astringency, as well as potent antioxidant effects [81] depending on their chemical structures.

In this current study, the range of polyphenol content (216.73–412.75 mg/L) is less than the value of ≈1800 mg/L for a typical commercial bottle of red wine reported by Mukamal et al. [87]. However, the amount of polyphenol content obtained in this study is higher than the values of 18–132 mg/L for fruit wines made from apples, strawberries, bilberries, and cowberries [88]. In other studies, wines made from cherries (1080 mg/L), red raspberries and blackcurrants (1050 mg/L), and blackcurrants and crowberries (1020 mg/L) were found to contain higher amounts of polyphenolic compounds [88]. This is an indication that the type of fruit is a function of the polyphenol content in wine. Importantly, polyphenols are components of wine, especially red wines, that do not exist in spirits but exist in low amounts in malt whiskey and beer. Its potential health benefits and bioactive properties, such as antioxidant effects, antimutagenic property, chelating effects on catalytic metals, and free radical scavenging effects, are the reason red wine has gained significant research attention for protecting against cardiovascular diseases (CVD) [79, 81, 89]. This was demonstrated in studies that showed an inverse correlation with overall mortality in people consuming red wine, but not in those consuming beer or spirits [90].

#### 3.1.7. Flavonoid Content

The flavonoid content of wine samples ranged from 50.87 to 126.53 mg/L (refer to Table 2). The foreign wines had a flavonoid content of 114.46 mg/L and 126.53 mg/L while the domestic wines had a flavonoid content of 50.87 mg/L and 88.13 mg/L. There were significant differences (*p* < 0.05) between the wine samples. Flavonoids are the most dominant group of polyphenolic compounds in red wine and are essential to the wine quality [79]. Among the flavonoids are the anthocyanins, flavanols or flavan-3-ols (catechin and epicatechin), and the flavonols (quercetin and myricetin), all of which have been linked to many health benefits [81, 91]. In this study, the foreign wines had higher flavonoid content (114.46–126.53 mg/L) compared to the domestic wines (50.87–88.13 mg/L). It is well known that the amount of flavonoids in red wine depends on the type of fruit and its variety, sun exposure, cultivation area, wine-making process and technique, and wine age [85]. However, the range of flavonoid content (50.87–126.53 mg/L) recorded in this work is lower than the values reported by He et al. [76] for hawthorn fruit.

Results showed that flavonoids constitute a major group of phenols in the wines. The domestic wines had a lower flavonoid/polyphenol relation compared to the foreign wines. This implies that 23.47–28.76% of polyphenols in domestic wines (DW2 and DW1, respectively) are from the flavonoid group while 29.72–30.63% of polyphenols in foreign wines (FW2 and FW1, respectively) are from the flavonoid group. Hodgson [92] stated that flavonoids are the main polyphenols present in red wine by weight and constitute about 80–90% of total polyphenols. The variations in the flavonoid/polyphenol relation could be attributed to differences in agronomic practice, environmental conditions, wine-making techniques, and fruit type used since they are primarily found in skins and seeds of fruits. According to Cheynier (2016), wine composition depends not only on the type of grape or other fruits used as raw material, which is influenced by varietal and agricultural factors, but also on the wine-making process, which determines the extraction of flavonoids into the liquid phase and their subsequent reactions.

Flavonoids have been shown to contribute to the organoleptic property in wines. They are responsible for the colour and astringency of red wines (Cheynier, 2016; [93]). Additionally, flavonoids are also directly linked to having health-promoting properties in red wine, especially for moderate wine consumers which is a result of its antioxidant property (Cheynier, 2016; [94]). Furthermore, the antioxidant capacity of flavonoids is probably due to the ability of their highly reactive hydroxyl group to directly scavenge free radicals [78]. This corroborates the report of Hodgson [92] that flavonoid-rich foods and beverages can have vascular health benefits.

#### 3.1.8. Tannin

The tannin content of wine samples ranged from 21.87 to 54.41 mg/L (refer to Table 2). The foreign wines had a tannin content of 49.22 mg/L and 54.41 mg/L while the domestic wines had a tannin content of 21.87 mg/L and 37.90 mg/L. There were significant differences (*p* < 0.05) between the wine samples. Tannins are important compounds in wines that influence the sensory characteristics of wine, especially red wine. In this study, foreign wines (49.22–54.41 mg/L) had significantly (*p* < 0.05) higher tannin content compared to domestic wines (21.87–37.90 mg/L). The variations in the tannin content of the wines could be a result of the difference in the type/variety of fruit used, vintage variation, and winemaking process. Specifically, tannins are polyphenolic compounds polymerized from flavonoids and nonflavonoid compounds. They are divided into hydrolysable tannins (polymers of nonflavonoids especially phenolic acids such as gallic acid and ellagic acid) and condensed tannins (polymers of flavonoids especially flavan-3-ols) [21, 82, 95].

In this current study, the range of the tannin content (21.87–54.41 mg/L) is less than 110–557 mg/L of the tannin content reported by Watrelot [96]. The lower tannin content obtained in the present study could be attributed to differences in the type of fruit used, the winemaking process, the concentration of polyphenolic compound, amount of anthocyanin content, ageing duration, and degree of polymerization [79, 86, 97]. According to Zhijing [98], the amount of anthocyanin in red wine greatly influences the degree of tannin polymerization due to anthocyanin-tannin interactions. Furthermore, tannins in red wine have been shown to come from different plant sources/parts such as fruit skins, seeds, stems, oak, and additives. However, studies have shown that fruit skin especially grape skin has a higher tannin concentration than tannins from other sources [99]. Also, the maceration steps with skins during winemaking increase the mass transfer of phenolics (tannins) from the skin to the wine [98].

Tannins are well known to be responsible for the bitter and astringency perception in wines. In other words, they are the textural components of wine responsible for giving (red) wines a defined structure or body [100]. Further, the taste perception of wine is also greatly influenced by other constituents in wine such as ethanol and pH. A higher level of ethanol ameliorates bitterness, while a lower pH level leads to a significant increase in astringency. Among polyphenolic compounds, tannins are the major components directly linked with astringency sensation. Astringency in red wine is a tactile sensation described as dryness, puckering, and tightening sensation perceived in the oral cavity (mouth). This is a result of interactions between tannins and proteins (glycoproteins of the mouth epithelium) in the oral cavity to form stable complexes, thereby causing a loss in the lubricating power of the saliva (Cheynier, 2016; [94]). As a polyphenolic compound, tannins have been shown to exhibit some health-promoting actions by acting as an antioxidant [86]. However, Zhijing et al. [98] indicated that tannin is one of the major phenolic compounds that contribute to free radical scavenging abilities. In addition to tannin's antioxidant capacity, its interaction with proteins in the mouth has been linked to promoting oral health, specifically curbing dental caries [94].

### 3.2. Variations in Antioxidant Properties of Wine Samples

The antioxidant properties of wine are presented in Table 3. It is well known that the antioxidant capacity of wine depends on its composition, concentration of active ingredients, and the test system conditions, which cannot be reported with a single method. Given this, the DPPH, FRAP, and TEAC assays were used to evaluate the antioxidant capacity of the wines. The antioxidant capacity varied significantly (*p* < 0.05) as DPPH of wines ranged from 28.16 to 57.04% and FRAP ranged from 2.38 to 4.92 mmol/L, while TEAC ranged from 14.53 to 27.02 mmol/L Trolox equivalent. Overall, the foreign wines had higher antioxidant properties compared to the domestic wines as FW1 recorded the highest value of the antioxidant property while DW1 had the least value. This trend was followed by the wine samples for all the assays evaluated. The variations in antioxidant capacities of the wines could be a result of differences in the types of fruits used, the processing method, and fermentation style, which could result in wines of different concentrations of polyphenols.

However, free radicals are a group of atoms, molecules, and/or compounds with electron-rich sites that are predominantly generated in food/biological systems, especially during cellular respiration [101, 102]. They have been frequently linked to oxidative stress and alteration of DNA, proteins, and lipids, thereby causing gene modification, neurodegenerative disease, cancer, cardiovascular disease, cell death, etc. [103], thus the need for antioxidants to scavenge these free radicals in wine.

Given this, the DPPH assay was used to evaluate the various wines' ability in scavenging DPPH free radicals. From the result, the foreign wines with higher DPPH scavenging activity compared to the domestic wines suggest that they can donate more hydrogen to free radicals, thus imparting higher wine stability and benefit to human health when consumed [76, 104]. The variations in antioxidant capacity in these wines in terms of DPPH could be a result of the flavonoid content and its concentration in the wine. Amongst polyphenolic compounds, flavonoids are known antioxidant agents with a reaction mechanism of scavenging free radicals by allowing themselves to be oxidized by the radical while the radical becomes more stable and less reactive. The reactivity of the flavonoid's hydroxyl group facilitates the inactivation of free radicals into a stable molecule or compound, which influences their activities [105, 106]. This corroborates the findings of Andrade and Fasolo [78] who reported a positive correlation between DPPH and flavonoid content.

Furthermore, FRAP assay determines the ability of the wines to reduce ferric to ferrous ions. According to the result, the higher reducing power of the foreign wines suggests that they possess high antioxidative compounds that can reduce Fe^3+^ to Fe^2+^. The reduction of ferric ions by wine antioxidants is important because they are responsible for auto-oxidation of the Fenton reaction which yields hydroxyl radical that can cause various life-threatening diseases [103]. Similar trends were reported by He et al. [76] in the study of Hawthorn wines fermented by wine yeast. On the other hand, the TEAC assay determines the ABTS radical scavenging ability of the wines. The wines had an appreciable ABTS radical scavenging ability but were lower than the values of 28–32 mmol/L of Trolox equivalent reported for fresh guava by Thaipong et al. [107]. Just like in DPPH and FRAP, the results show that the antioxidants in the wines can neutralize radical cation ABTS^•+^ either by direct reduction via electron donation or by radical quenching through hydrogen atom donation [108]. Besides flavonoids, there is also existing evidence indicating anthocyanins' positive therapeutic properties by acting as an antioxidant. However, phenolic compounds are generally responsible for antioxidant properties in wines. Although their various structural characteristics determine to a great extent the different levels of antioxidant activity [78], the pH of the wine is also very vital. This corroborates the findings of Prior et al. [109] who reported that the antioxidant structure and pH of the medium determine the balance between the reaction mechanisms of antioxidant capacity. This suggests that a lower pH value enhances wine's antioxidant capacity. To reiterate, this study has shown that foreign wines had higher antioxidant properties compared to domestic wines. In addition, there appears to be a correlation between antioxidant properties and polyphenolic compounds as the wine samples with a higher concentration of polyphenolic compound (refer to Table 2) had higher antioxidant capacity.

### 3.3. Variations in the Colour of Wine Samples

The chromatic characteristics of the wine samples are shown in Table 4. The wines varied significantly (*p* < 0.05) amongst each other as *L*^∗^ ranged from 39.12 to 52.02, *a*^∗^ ranged from 22.37 to 54.23, and *b*^∗^ ranged from 19.76 to 39.37. Whilst parameter *a*^∗^ takes negative and positive values for greenish and reddish colours, respectively, parameter *b*^∗^ takes negative values for bluish colours and positive values for yellowish ones. On the other hand, parameter *L*^∗^ measures the luminosity, which considers colour as a member of the grey scale, between black and white [110]. Results show that the domestic wines (DW2 and DW1) had the highest and lowest *L*^∗^, *a*^∗^, and *b*^∗^ values, while the foreign wines (FW2 and FW1) lie in between the two extreme points of the colour spaces. The variations in the wine CIELab colour space values can be a result of differences in the type of fruits used in the production of wine. Though grapes are well known as the principal fruit for wine production in the temperate region, there are still slight variations in wine colour produced in those regions due to varietal differences. On the contrary, grapes are not used in wine production in the tropics, and as a result, synthetic or natural colourants are added to white wines produced from tropical fruits to meet the demand for red wine [111]. More so, the use of natural pigments derived from plants for colouring beverages is now a commercial reality due to the adverse effects of utilizing synthetic colours in food applications [33, 46]. This was demonstrated in a recent study on the evaluation of the fruit wine quality from blends of pawpaw, date fruit, and roselle by Ofili [112]. Notably, the proportion of *L*^∗^, *a*^∗^, and *b*^∗^ colour values is an indication of the magnitude of the wine colour characteristic which therefore implies that the DW2 appeared brighter, more reddish, and more yellowish compared to other wines while DW1 was darker, less reddish, and less yellowish than the other wines. The wide variations in domestic wines compared to foreign wines suggest the lack of regulatory standards and limits for colourants used in producing red wines from tropical fruits.

**Figure 2:**
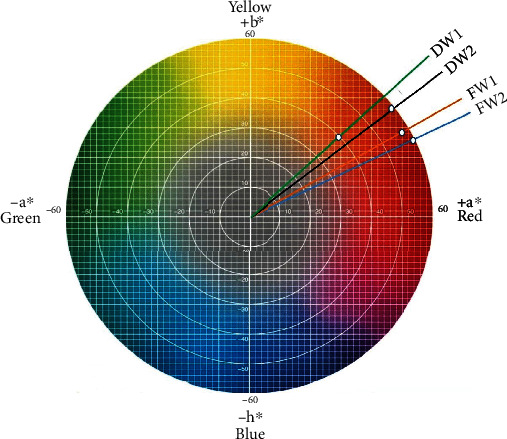
Wine colour spectrum.

Furthermore, the hue angle and chroma values of wine samples ranged from 29.93°–41.46° and 29.85–67.01, respectively (refer to Table 4). Whilst the hue angle is the qualitative attribute of colour which reveals the human perception of colour, chroma is the quantitative attribute of colour which measures the magnitude of the difference of a hue in comparison. In other words, chroma describes the dullness and vividness of a colour [113]. Generally, results show that the hue angle is less than 90° (i.e., 29.93°–41.64°) which implies less yellowish and indicates a red colour spectrum in the colour space of the visible region in the opponent colour chart. However, the dominance of hue is determined by the chroma which is a representation of the wine's colour intensity perceived by humans [33] as shown in Figure 2.

The magnitude of wine's chroma in this study corresponds to their degree of saturation ranging from vivid to dull. It could also be observed that the chroma values of domestic wine possessed both dullness (DW1: 29.85) and vividness (DW2: 67.01) while the foreign wines fell in between the two extremes. This is also indicative of a lack of acceptable standards and limits when utilizing colourants in domestic wine production, though wine colour has a wide range of variations. The variations in hue and chroma values could be due to the concentration of anthocyanins in the wine samples. Anthocyanins are polyphenols that are responsible for red pigmentation in red wines [114]. Interestingly, the differences in grape sources and varieties could be responsible for the different levels of red pigmentation in the wine, especially the foreign wines. For the domestic wines, artificial or natural colourants such as roselle (*Hibiscus sabdariffa*) might have been used to impart a red colour to the wine. The anthocyanins from roselle calyces are known to produce brilliant red colourings in beverages [114]. Besides fruit (grape) type and varieties, vintage year, and ageing period, anthocyanins and the overall colour of wine are influenced by some other factors such as pH, SO_2_, light, heat, metals, and copigmentation [114]. Additionally, certain sugars have been reported to influence the colour intensity of anthocyanins in wines. Possibly, the combined effect of sugar and acids at various proportions may have influenced the colour of wines differently. The importance of wine colour as an indicator of quality cannot be over emphasized as it is the first quality characteristic perceived by the consumer [46].

The colour differences (Δ*L*^∗^, Δ*a*^∗^, and Δ*b*^∗^) of the wine samples are shown in Table 5. With regard to the foreign wines, FW2 appears to be slightly darker, more reddish, and more yellowish compared to FW1. Concerning the domestic wines, DW2 appears to be brighter, more reddish, and more yellowish to a greater extent compared to DW1. For the colour difference between foreign wines and domestic wines, results show that foreign wines appeared to be slightly brighter, more reddish, and less yellowish (or more bluish). However, the total colour difference (Δ*E*) tends to buttress the facts already established by the colour differences of the individual colour coordinates. In this study, Δ*E* measures the magnitude of colour variation between the two foreign wines, the two domestic wines, and all the foreign wines and domestic wines. A value in Δ*E* proves the existence of a colour difference [33]; hence, the larger the value of Δ*E*, the greater the intensity of the colour difference. Results show that the Δ*E* between the two foreign wines is significantly lower (*p* < 0.05) compared to the two domestic wines. Similarly, the Δ*E* between the foreign wines and domestic wines shows that colour variation of a noticeable margin exists. These colour differences in wines were expected because of differences in types of fruits used, winemaking process, fermentation method, additive and colourants used, ageing period, etc. Ageing of wine has been demonstrated to change the organoleptic properties of wine, especially colour and taste, due to oxidation [115].

### 3.4. Sensory Characteristics of Wine Samples

**Figure 3:**
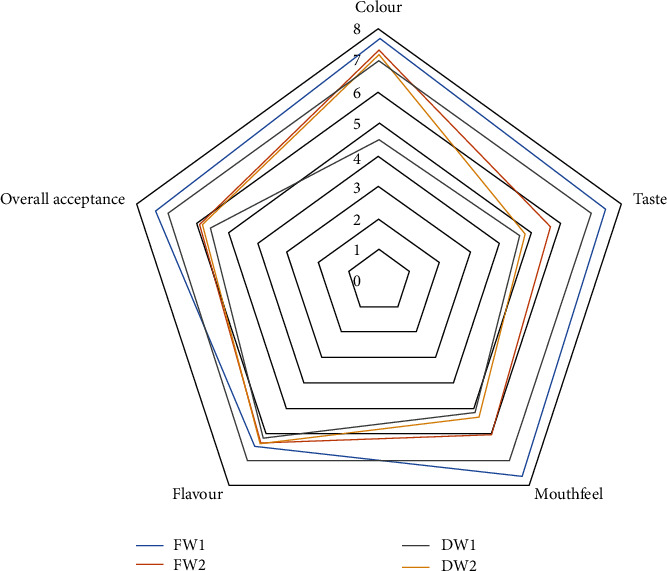
Sensory perception of foreign and domestic wines.

**Figure 4:**
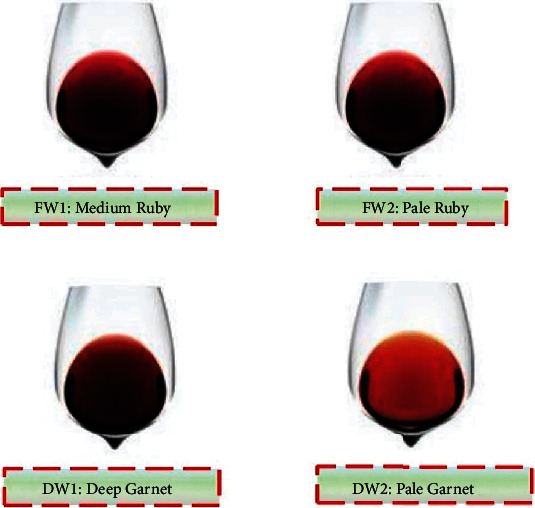
Colour difference between foreign and domestic wines (adapted from [117]).

The sensory properties (colour, taste, mouthfeel, flavour, and overall acceptability) of wines varied significantly (*p* < 0.05) (refer to Figure 3). Based on the hedonic scale, the panelists viewed the colour of wines (4.50–7.67) as medium ruby (FW1), pale ruby (FW2), pale garnet (DW2), and deep garnet (DW1) from the wine colour chart (Figure 4). The panelists considered DW1 as disliked slightly while DW2, FW1, and FW2 were liked moderately. The colour variations in the wine samples may be due to varying concentrations of anthocyanins. Additionally, the fruit type, vintage year, fermentation process, and ageing period, as well as different levels of sugars and acids, can influence the wine. Studies have shown that the red pigment from anthocyanins is intensified and highly retained at lower pH (acidic medium). Also, certain sugars have been shown to influence the colour intensity of anthocyanins in wines.

The panelists adjudged the taste of domestic wines (DW1 and DW2) as disliked slightly while the foreign wines, FW2, were neither liked nor disliked and FW1 was liked moderately. The variations in the taste of different wine samples could be a result of different concentrations of organic acids and residual sugars. The organic acid profile of the wine is linked to some sensory descriptors such as being fresh, sour, or metallic [56]. However, the interaction between acids and residual sugars in wine can also influence its taste perception. This corroborates the findings of Mato et al. [116] that desirable acidity is a function of wine sweetness, which is mostly but uniquely derived from residual sugars. More residual sugar can be necessary with such a wine to balance the sourness and sweetness to obtain good flavours and tastes. This implies that other wine constituents such as tannins—a known contributor to bitterness—can also impact its sensory properties. Further, the taste perception of wine is also greatly influenced by other constituents in wine such as ethanol and pH. A higher level of ethanol enhances bitterness whereas a lower pH level leads to a significant increase in astringency (Cheynier, 2016). Bitterness perception is a taste recognition mediated by taste buds present in the tongue papillae. It is usually perceived in wines with a large number of polyphenols especially flavan-3-ols and their polymers [82].

Furthermore, the panelists assessed the mouthfeel of domestic wines as neither liked nor disliked while the foreign wines, FW2, were slightly liked and FW1 was moderately liked (refer to Figure 4). Though the domestic wines appeared relatively flat or flappy compared to the foreign wines, the low rating of mouthfeel by panelists could be a result of astringency sensation. Astringency is an oral sensation involving dryness, shrinking, and puckering of epithelium owed to exposure to tannins. This is a result of the corresponding precipitation of complexes when tannins interact with salivary proteins [82]. The variations in the phenolic profile of the wine may be responsible for the differences in the mouthfeel perception of wine by the panelists.

The wine flavour, on the other hand, was considered to be slightly liked by the panelists as it ranged from 6.17 to 6.50. Specifically, the flavour of wine is characterized by a volatile compound profile [118–120] influenced principally by yeast metabolism during fermentation. The differences in flavour of wine samples may occur with fermentation by-products, such as aroma-active esters, higher alcohols, aldehydes, and phenolic compounds [46, 121, 122]. This corroborates with the attestation of pungency in the foreign wines by the panelists, as foreign wines tend to have a higher concentration of alcohol and polyphenolic compounds compared to domestic wines.

The overall acceptance herein suggests that the sensory properties of wine might influence consumer preference for these wine samples, as FW1 was liked moderately and FW2, DW1, & DW2 were neither liked nor disliked. Overall, the sensory properties show that organic acids, residual sugars, and phenolic compounds directly influence the organoleptic character of wines.

## 4. Conclusion

In this study, the physicochemical, antioxidant, and sensory properties of foreign and domestic wines were evaluated as markers of red wine quality and authenticity. The foreign wines had significantly higher total titratable acidity, brix, alcohol, and polyphenol content while the domestic wines had significantly higher pH and total sugar content. The presence of phenolic compounds in the wines confirmed their authenticity. Specifically, the foreign wines exhibited significant higher (*p* < 0.05) antioxidant capacity compared to the domestic wines. The ability of the wines to exhibit antioxidant properties is an indication of the quality that the substrates or fruits (raw material) passed through fermentation. Although the hue angle (27.68°–41.46°) of the wines indicated a red colour spectrum in the colour space of the visible region of the opponent colour chart, the total colour difference between the foreign and domestic wines was significant. Besides the sensory profile resembling in flavour, the overall acceptability of DW1, DW2, and FW2 was neither liked nor disliked, as FW1 was liked moderately by the panelists. The type of fruit, fermentation method, winemaking process, and ageing period influence the wine quality. Overall, the wines showed a considerable level of authenticity and different levels of quality according to their varying concentration of organic acids, residual sugars, and phenolic compounds. In other words, wine quality and authenticity are a function of the relationship between wine's organic acids, sugars, and phenolic compounds. More research is advocated on the use of natural colourants from plants in producing domestic red wine from different tropical fruits since grapes (temperate fruit) do not grow in the tropics. To establish consistency and product uniformity, it is recommended that there should be regulated standards and limits on the amount of natural colourants required in domestic red wine made from tropical fruits. To minimize adulteration and fraud related to domestic red wine, it is recommended that a rapid analytical tool be developed for the on-site evaluation of the wine quality by regulatory agencies.

## Figures and Tables

**Table 1:** Variations in pH, total titratable acidity, brix, reducing sugar, and total sugar of wine samples.

Sample	pH	TTA (%)	Brix (g/100 g)	Total sugar (%)
FW1	3.51^d^ ± 0.14	0.83^a^ ± 0.25	8.04^a^ ± 0.82	6.13^d^ ± 0.22
FW2	3.87^c^ ± 0.07	0.76^b^ ± 0.03	6.98^b^ ± 0.11	7.84^c^ ± 0.38
DW1	4.03^b^ ± 0.01	0.68^c^ ± 0.13	5.08^c^ ± 0.61	8.60^b^ ± 0.12
DW2	4.16^a^ ± 0.21	0.56^d^ ± 0.28	4.22^d^ ± 0.21	9.27^a^ ± 0.84

^a,b^Significantly different (*p* < 0.05). FW1: foreign wine—Spanish; FW2: foreign wine—French; DW1: domestic wine—Owerri; DW2: domestic wine—Aba.

**Table 2:** Variations in alcohol, polyphenol, flavonoid, and tannin content of wine samples.

Sample	Alcohol (% ABV)	Polyphenol (mg/L)	Flavonoid (mg/L)	Tannin (mg/L)
FW1	15.21^a^ ± 0.28	412.75^a^ ± 0.54	126.53^a^ ± 0.04	54.41^a^ ± 0.00
FW2	14.44^b^ ± 0.92	385.13^b^ ± 0.42	114.46^b^ ± 0.42	49.22^b^ ± 0.01
DW1	8.52^d^ ± 0.27	306.42^c^ ± 0.82	88.13^d^ ± 0.28	37.90^c^ ± 0.01
DW2	10.68^c^ ± 0.56	216.73^d^ ± 0.35	50.87^c^ ± 0.13	21.87^d^ ± 0.00

^a,b^Significantly different (*p* < 0.05). FW1: foreign wine—Spanish; FW2: foreign wine—French; DW1: domestic wine—Owerri; DW2: domestic wine—Aba.

**Table 3:** Antioxidant properties of wine samples.

Sample	DPPH (%)	FRAP (mmol/L)	TEAC (mmol/L)
FW1	57.04^a^ ± 0.22	4.92^a^ ± 0.31	27.02^a^ ± 0.14
FW2	42.34^b^ ± 0.41	4.33^b^ ± 0.82	18.96^b^ ± 0.21
DW1	28.16^d^ ± 0.16	2.38^d^ ± 0.28	14.53^d^ ± 0.02
DW2	36.50^c^ ± 0.35	3.18^c^ ± 0.42	16.34^c^ ± 0.49

^a,b^Significantly different (*p* < 0.05). FW1: foreign wine—Spanish; FW2: foreign wine—French; DW1: domestic wine—Owerri; DW2: domestic wine—Aba; TEAC: Trolox equivalent antioxidant capacity; FRAP: ferric reducing antioxidant power; DPPH: diphenyl-1-picrylhydrazyl.

**Table 4:** Chromatic characteristics of wine in CIELab colour space.

Sample	*L* ^∗^	*a* ^∗^	*b* ^∗^	*H* ^∗^	*C* ^∗^
FW1	48.51^b^ ± 0.57	43.13^b^ ± 0.33	24.83^c^ ± 0.21	29.93°	49.77
FW2	47.87^b^ ± 0.13	51.87^a^ ± 0.25	27.20^b^ ± 0.12	27.68°	58.57
DW1	39.12^c^ ± 0.12	22.37^c^ ± 0.18	19.76^d^ ± 0.34	41.46°	29.85
DW2	52.02^a^ ± 0.46	54.23^a^ ± 0.54	39.37^a^ ± 0.43	35.99°	67.01

^a,b^Significantly different (*p* < 0.05). FW1: foreign wine—Spanish; FW2: foreign wine—French; DW1: domestic wine—Owerri; DW2: domestic wine—Aba.

**Table 5:** Colour difference of wine samples.

Sample	Δ*L*^∗^	Δ*a*^∗^	Δ*b*^∗^	Δ*E*^∗^
FW2-FW1 (foreign)	−0.64	8.74	2.37	9.08
DW2-DW1 (domestic)	12.90	31.86	19.61	39.57
FW1,2-DW1,2 (foreign-domestic)	2.62	9.20	−3.55	10.20

FW1: foreign wine—Spanish; FW2: foreign wine—French; DW1: domestic wine—Owerri; DW2: domestic wine—Aba; FW1,2: all foreign wines; DW1,2: all domestic wines.

## Data Availability

Data will be made available upon request.
